# The anti-anxiety drug lorazepam changes implicit behaviors but not explicit evaluations of sense of agency under authoritative pressure: A functional magnetic resonance imaging study

**DOI:** 10.3389/fpsyg.2022.991357

**Published:** 2022-11-21

**Authors:** Chenyi Chen, Róger Marcelo Martínez, Yu-Chun Chen, Yang-Teng Fan, Yawei Cheng

**Affiliations:** ^1^Department of Physical Medicine and Rehabilitation, National Yang Ming Chiao Tung University Hospital, Yilan, Taiwan; ^2^Graduate Institute of Injury Prevention and Control, College of Public Health, Taipei Medical University, Taipei, Taiwan; ^3^Research Center of Brain and Consciousness, Shuang-Ho Hospital, Taipei Medical University, Taipei, Taiwan; ^4^Graduate Institute of Mind, Brain and Consciousness, College of Humanities and Social Sciences, Taipei, Taiwan; ^5^Psychiatric Research Center, Wan Fang Hospital, Taipei Medical University, Taipei, Taiwan; ^6^School of Psychological Sciences, National Autonomous University of Honduras, Tegucigalpa, Honduras; ^7^Department of Physical Education, National Taiwan University of Sport, Taichung, Taiwan; ^8^Graduate Institute of Medicine, Yuan Ze University, Taoyuan City, Taiwan; ^9^Institute of Neuroscience, National Yang Ming Chiao Tung University, Taipei, Taiwan; ^10^Department of Education and Research, Taipei City Hospital, Taipei, Taiwan

**Keywords:** lorazepam, coercion, sense of agency (SoA), hippocampus, intervention

## Abstract

Previous research on coercion has neglected the fact that agents under authoritative pressure may also suffer from coercive power, which can trigger anxiety-like emotional negativity on its victims. Furthermore, high levels of neuroticism and/or anxiety have been found to be associated with the compliance of various forms of social pressure. In this study, we investigate the effects of the anxiolytic GABA_*A*_ (gamma-Aminobutyric acid) modulator, lorazepam, on behavioral and neural responses to coercive power. Here, we applied a virtual obedience to authority paradigm alongside lorazepam administration (versus placebo), and during functional magnetic resonance imaging scanning. Our results show that lorazepam administration exerted differential effects on the reaction times (RTs) when initiating harming versus helping behaviors, with longer harming RTs compared to helping RTs, despite comparable subjective ratings regarding perceived coercion. Coercive harming significantly increased activity in the amygdala, hippocampus, orbitofrontal cortex, and dorsolateral prefrontal cortex (dlPFC). Lorazepam administration decreased amygdala and hippocampus activity, but increased dlPFC and right temporoparietal junction activations. The lower activity in the hippocampus predicted higher ratings for perceived coercion. Furthermore, lorazepam significantly decreased the functional connectivity of the hippocampus with the dlPFC during coercive harming. In conclusion, we provide evidence –by incorporating multimodal indices, including neuroimaging, neuropharmacological interventions, and behavioral assessments– to posit that the GABA_*A*_ agonist, lorazepam, might aid as a possible intervention in service of coping strategies against coercion.

## Introduction

Throughout history, it has been well documented that people are enabled to perform the most vilest and unscrupulous actions against other human beings when under the pressure of an authority figure (Arendt, 1994). Furthermore, Stanley Milgram’s experiments were, allegedly, able to confirm such observations (Milgram, 1963, 1965), as the participants were ready to comply with the experimenters’ coercive orders to inflict intolerable harm (in this case in the form of electric shocks) to another participant just for the benefit of the experiment itself. However, this view has recently been questioned (Perry, 2013). While past experimental studies on coercion have skewed toward dissembling manipulation or participants’ obedience levels, they have neglected the fact that agents under coercive power are also victims. What Milgram’s experiments failed to take into consideration, and what remains invisible in historical accounts where coercion has played an important role, is that in real-life situations agents under authoritative pressure are also victims of the most serious and insidious forms of coercive power, subjected through immense amounts of pressure to harm themselves and/or those who they love –e.g., victims of domestic and/or intimate partner violence and abuse (Frank and Rodowski, 1999; Myhill and Hohl, 2019); consequently exhibiting differences in negative emotional responses to their actions, such as anxiety or remorse. This important issue remains to be addressed at the behavioral and neural levels, as well as potential interventional approaches as to turn over the toll of coercion.

Furthermore, neuroticism and negative affect have been found to influence the reluctance with which an individual engages in destructive obedience in the Milgram Paradigm (Zeigler-Hill et al., 2013). Individuals who are more likely than others to avoid risk (Maner et al., 2007) and act in accordance with distinct ways of social pressure (Drake, 2010), and thus adhere to rules (Allik and McCrae, 2004), tend to have higher levels of neuroticism, as the negative affect/anxiety in which said individuals incur makes them more prone to avoid uncertainty (Zeigler-Hill et al., 2013). Hence individuals under coercive control frequently suffer from anxiety. On one hand, previous neuroscientific research addressing coercion has shown that anxiety and/or fear congeals cognitive resources and hampers cognitive functions, in such a way that the participants who obeyed orders did not subjectively experience their actions as voluntary ones, but rather as passive movements devoid of any sense of agency (Caspar et al., 2016, 2018, 2020a,2020b,^2021^). At a neural level, this was observed as a reduction in the auditory N1 ERP component amplitude when the subject was under coercion, relative to the condition where he could choose freely to perform an action; thus, the brain engaging in a decrement of sensory processing in anticipation of the action outcomes, and which, again, translates into perceiving the repercussions of actions executed under coercive pressure as if they had been triggered passively (Caspar et al., 2016, 2021). On the other hand, studies exploring the neural origin of the N1 component, did so by isolating glutamatergic receptor function through micro-injections of the somatosensory barrel cortex (S1BF) in anesthetized rats with sub-convulsive concentrations of the competitive gamma-aminobutyric acid (GABA_*A*_) receptor antagonist bicuculline methiodide (BMI) (Krishek et al., 1996; Ueno et al., 1997; Jones and Barth, 2002; Johnston, 2013); and with the results showing that the greater the suppression of inhibition by the BMI, the broader and larger the N1 pulse width and amplitude became, respectively (Bruyns-Haylett et al., 2017). The anxiolytic drug lorazepam is a high-potency 3-hydroxy benzodiazepine prescribed for the relief of anxious symptomatology (Gould et al., 1997), as it enhances GABA release in the brain by binding to the GABA receptors. Interestingly, Perkins et al. (2013) demonstrated that lorazepam incurred in a dose-dependent increase in the participants’ willingness to endorse responses that directly harm others in moral-personal dilemmas, regardless of whether the motivation for those harmful acts is selfish or utilitarian. This seemingly unrelated study seems to gain even greater relevance when we take into account the research done in the N1 ERP component and the GABAergic system, in the sense that Perkins et al.’s (2013) work would, hypothetically, situate itself between the notion of sense of agency and the GABA-induced auditory N1 component.

Before going further, it is important to note that “coercion” itself cannot be studied directly –as if in a real-life situation– in an experimental setting due to ethical concerns. Strictly speaking, the term “coercion” encompasses actions that make use of force or violence, either directly or indirectly (through the use of threats), in order to induce an individual into doing something to which they have not given their full or partial consent. Consequently, in the present study, we use the notion of “coercion” to describe an experimental condition in which agents execute orders which lead them to inflict harm to another individual. In a recent study, we demonstrated that the effect of social coercion on the experience of agency and responsibility was modulated by individual variability in response to coercive power (Cheng et al., 2021a). In the virtual obedience paradigm, participants watching the first image of a morally laden scenario mini clip were forced (ordered by textual instructions) to press a handheld button in order to initiate the successive actions that carry different moral consequences, including harming others, along with visual feedback of such moral scenarios. The handheld button portion was designed not only to provide aid in engaging and taking the perspective of the virtual agent, but also to measure the participants’ willingness to conform to the instructions of an authority figure and obey the coercive order to commit harming actions. Participants tend to take longer RTs to initiate harming relative to neutral actions, as such, the RTs to initiate actions could be a valuable measure of implicit behaviors in response to coercive power.

To understand a potential intervention that may lead people to counteract the effects of coercive violence, we investigate the behavioral and neural effects of the anxiolytic drug lorazepam on the virtual obedience paradigm. Nevertheless, and contrary to the research mentioned above, the present study makes use of functional magnetic resonance imaging (fMRI). This is, firstly, due to the amygdala being a key brain structure responsible for salience detection in the environment, including threats (Davis and Whalen, 2001); thus, we consider fMRI to be a better approach for measuring amygdala reactivity. It is also important to note that the extent to which the amygdala reacts in response to threatening stimuli has been linked to anxiety (Etkin et al., 2004; Chenyi Yu-Chun Chen et al., 2019), and which constitutes a secondary reason for using lorazepam, as its administration should –hypothetically– modulate said anxiogenic response, in addition to the willingness of the participants to commit (or not) certain kinds of moral acts. Specifically, our primary hypothesis is that lorazepam would significantly enhance our participants’ willingness to stand against coercive power, as the evidence regarding the relationship between the sense of agency, the N1 component, and its GABAergic system-related origin, may point toward this outcome. Furthermore, and as an exploratory extension, we sought to examine whether these two types of moral coercion (harming and helping others) would be differentially affected by lorazepam. To avoid the unwanted slowing effect on the behavioral measures, we used a 0.5 mg dosage of lorazepam, and which was previously shown to exert its effect on both behavioral and neural modulations without producing significant sedation (Arce et al., 2006; Diaper et al., 2012; Gagnon et al., 2019).

## Results

### Reaction times and subjective ratings

Within each participant, the mean RTs were firstly calculated and represented the reaction speed of the six different conditions (placebo harming, placebo helping, placebo neutral, lorazepam harming, lorazepam helping, lorazepam neutral) (Table 1). Due to these mean RTs not being normally distributed across participants, the mean RTs were base-10 LOG-transformed for further analyses (please see Supplementary Figure 2 and Supplementary Tables 2,3 for the results of sensitivity tests, normality plots and tests, and descriptive statistics of RTs in each condition).

**TABLE 1:** Descriptive statistics for raw and log_10_ RTs after placebo and lorazepam administration.

	Placebo	Lorazepam
Raw RTs (ms)	Mean ± SD	Mean ± SD
Harming	1062.237 ± 507.222	1127.344 ± 571.368
Helping	1042.104 ± 558.6	1032.073 ± 509.664
Neutral	1188.273 ± 852.63	1129.498 ± 526.734
**Log_**10**_ RTs**		
Harming	2.988 ± 0.191	3.015 ± 0.194
Helping	2.975 ± 0.201	2.958 ± 0.186
Neutral	3.024 ± 0.199	3.017 ± 0.185

**FIGURE 1:**
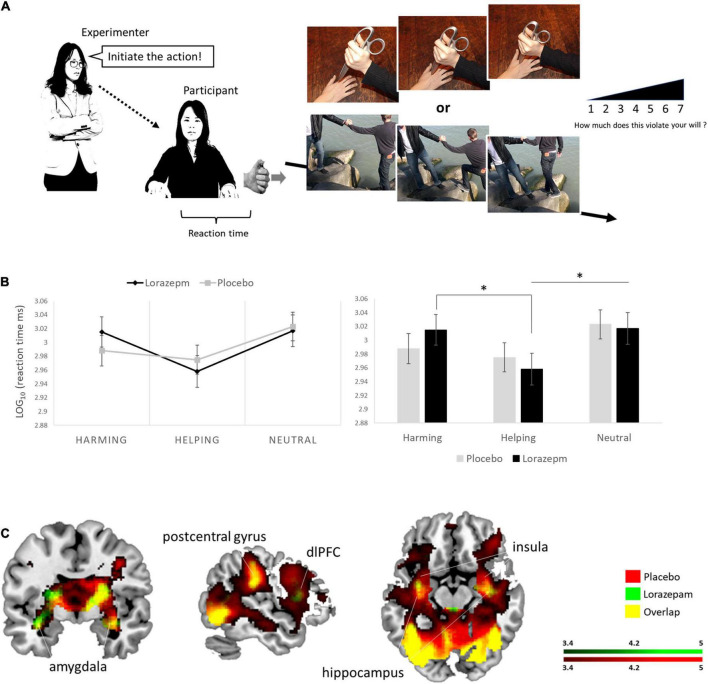
Experimental If permissions have been obtained, please confirm and we will add the following to your Ethics statement:Written informed consent was obtained from the individual*s* for the publication of any identifiable images or data included in this article.setup and lorazepam impact on moral behaviors under coercive pressure. **(A)** Schematic representation of the virtual obedience paradigm. The experimenter ordered the participant via textual instruction to initiate moral behaviors by pressing a handheld button along with visual feedback of such moral scenarios. **(B)** A 2 (administration: placebo vs. lorazepam) × 3 (scenario: harming vs. helping vs. neutral) repeated ANOVA on LOG-RTs revealed an interaction between administration and scenario (*F*_2,152_ = 3.64, *P* = 0.03, η^2^ = 0.05). Follow-up analyses indicated that the administration effect (lorazepam vs. placebo) had opposite directions depending on the factor of scenario, where lorazepam administration prolonged harming RTs, while shortening helping RTs, and did not have any effect on neutral RTs (harming: 3.02 ± 0.02 vs. 2.99 ± 0.02; helping: 2.96 ± 0.02 vs. 2.98 ± 0.02; neutral: 3.02 ± 0.02 vs. 3.02 ± 0.02). Harming and helping LOG-RTs differed significantly after lorazepam administration (*t*_76_ = 4.86, *P* < 0.001, Cohen’s *d* = 0.554), so did the neural and helping LOG-RTs (*t*_76_ = 4.19, *P* < 0.001, Cohen’s *d* = 0.4779). **(C)** Lorazepam impact on the whole-brain hemodynamic responses to coercive harming. Results from the whole-brain contrast thresholded at *P* < 0.001 and cluster extent *k* > 10 for viewing. *Represent the statistically significant. Informed consent was obtained from all subjects for publication of identifying information/images in an online open-access publication.

A 2 (administration: placebo vs. lorazepam) x 3 (scenario: harming vs. helping vs. neutral) repeated ANOVA on LOG-RTs revealed an interaction between administration and scenario (*F*_2,152_ = 3.64, *P* = 0.03, η^2^ = 0.05) as well as a main effect of scenario (*F*_2,152_ = 18.19, *P* < 0.001, η^2^ = 0.19). Follow-up analyses indicated that the harming and helping LOG-RTs differed significantly after lorazepam administration (*t*_76_ = 4.86, *P* < 0.001, Cohen’s *d* = 0.554), whereas they were comparable after placebo (*t*_76_ = 1.62, *P* = 0.11, Cohen’s *d* = 0.1851). The administration effect (lorazepam vs. placebo) had opposite directions depending on the factor of scenario, where lorazepam administration prolonged harming RTs, while shortening helping RTs, and did not have any effect on neutral RTs (harming: 3.02 ± 0.02 vs. 2.99 ± 0.02; helping: 2.96 ± 0.02 vs. 2.98 ± 0.02; neutral: 3.02 ± 0.02 vs. 3.02 ± 0.02). As a consequence of that, the harming and helping LOG-RTs differed significantly after lorazepam administration (*t*_76_ = 4.86, *Bonferroni-corrected P* < 0.05, Cohen’s *d* = 0.554), similar results were obtained with the neutral and helping LOG-RTs (*t*_76_ = 4.19, *Bonferroni-corrected P* < 0.05, Cohen’s *d* = 0.4779) (Figure 1B).

To isolate the sedation effect (slowing) of lorazepam, the differential LOG-RTs for helping/harming actions were derived from subtracting participant’s LOG-RTs in the neutral condition from LOG-RTs in the helping or harming condition. The differential LOG-RTs subject to a 2 (administration: lorazepam vs. placebo) × 2 (scenario: harming vs. helping) repeated ANOVA further confirmed the unique interaction between administration and scenario (*F*_1,76_ = 9.4, *P* = 0.003, η^2^ = 0.11) in addition to a main effect of scenario (*F*_1,76_ = 23.61, *P* < 0.001, η^2^ = 0.19) that showed shorter RTs in helping behaviors (helping vs. harming: –0.054 ± 0.011 vs. –0.018 ± 0.009, mean ± SE). Follow-up analyses indicated that the administration effect (lorazepam vs. placebo) had opposite directions depending on the factor of scenario (harming: –0.002 ± 0.015 vs. –0.035 ± 0.012, *t*_76_ = 1.715, *uncorrected P* = 0.09, Cohen’s *d* = 0.196; helping: –0.059 ± 0.014 vs. –0.049 ± 0.013). Here, the descriptive statistics for the *post hoc* tests were conducted and supplemented for the sake of revealing how the interactions functioned. Harming and helping LOG-RTs differed significantly after lorazepam administration (*t*_76_ = 4.86, *Bonferroni-corrected P* < 0.05, Cohen’s *d* = 0.554), whereas they were comparable after placebo (*t*_76_ = 1.625, *Bonferroni-corrected P* > 0.05, Cohen’s *d* = 0.1851). The descriptive statistics for the *post hoc* tests were conducted and supplemented for the sake of revealing how the interaction functioned. Complementary non-parametric analyses using raw RTs were reported in the Supplementary material.

The subjective ratings of coercion, derived from the violation of free will, were subject to a 2 (administration: lorazepam vs. placebo) × 2 (scenario: harming vs. helping) repeated ANOVA. There was a main effect of scenario (*F*_1,76_ = 49.19, *P* < 0.001, η^2^ = 0.39), indicating that, under coercion, participants were less willing (i.e., more self-reported violation to their own will) to do harming (5.15 ± 0.13) than to helping (4.53 ± 0.14). Neither the administration (*F*_1,76_ = 0.15, *P* = 0.70) nor its interaction with scenario (*F*_1,76_ = 0.13, *P* = 0.72) reached significance.

In order to increase the potential practical implication of this study, we further checked the number of participants who exhibited the same pattern reported at the group level. Regarding to the RTs, 56% of participants exhibited the same pattern reported at the group level in which RTs varied as a function of moral valence (i.e., harming RTs > helping RTs), whereas 72% of participants exhibited this pattern in the lorazepam condition. Regarding to the subjective ratings, while 70% of participants showed patterns of moral valence (i.e., harming ratings > helping ratings) in the placebo condition, 77% of participants showed this pattern in the lorazepam condition.

### Functional magnetic resonance imaging results

Table 2 lists the brain regions showing a significant hemodynamic change to coercive harming and helping after placebo and lorazepam administration. In response to coercive harming (vs. neutral), both lorazepam and placebo administration showed activations in the amygdala, hippocampus, putamen, anterior insula, temporal pole, thalamus, orbitofrontal cortex, dorsomedial prefrontal cortex, and dorsolateral prefrontal cortex (dlPFC) (Figure 1C). Regions with greater activity during coercive harming vs. helping showing significant hemodynamic increase were the anterior insula, amygdala, hippocampus, orbitofrontal cortex, dorsomedial prefrontal cortex, and dlPFC. On the other hand, regions with greater activity during coercive helping vs. harming showing significant hemodynamic increase were the dlPFC, rTPJ, and posterior cingulate.

**TABLE 2:** Brain regions showing significant BOLD activities to coercive harming and helping after placebo and lorazepam administration.

Brain regions	Side	MNI coordinates			*t*
		*x*	*Y*	*Z*	
**Coercive harming vs. Neutral**					
Temporal pole	R	48	8	–24	4.03
vmPFC	–	0	50	–20	3.69
Amygdala	R	28	–2	–20	5.89
Amygdala	L	–30	–4	–14	5.06
Anterior insula	R	28	16	–18	4.29
Anterior insula	L	–28	16	–18	4.15
Hippocampus	R	32	–10	–14	6.77
Hippocampus	L	–32	–10	–14	6.34
Orbitofrontal cortex	R	40	36	–14	3.51
Orbitofrontal cortex	L	–38	32	–8	4.12
Putamen	R	26	4	–4	5.34
Putamen	L	–30	4	–4	5.95
Thalamus	L	–20	–28	0	8.76
Thalamus	R	20	–20	2	8.16
Middle occipital gyrus	R	36	–88	0	10.43
dlPFC	L	–46	18	14	5.41
dlPFC	R	54	20	28	4.18
dmPFC	–	0	62	24	6.64
Supramarginal gyrus	L	–54	–26	30	8.19
Postcentral gyrus	R	56	–22	30	7.13
Midcingulate gyrus	–	0	0	38	3.69
Precentral gyrus	L	–14	–12	74	3.31
**Coercive helping vs. Neutral**					
Temporal pole	R	50	10	–24	3.83
Amygdala	L	–30	–4	–20	3.14
Insula	L	–36	–14	–4	3.92
Thalamus	R	22	–24	–2	8.57
Thalamus	L	–16	–28	2	7.34
Inferior frontal gyrus	L	–52	40	4	3.53
Middle occipital gyrus	R	28	–90	6	12.3
rTPJ	R	64	–44	12	3.35
Cingulate gyrus	R	12	2	28	3.73
dlPFC	R	50	22	32	4.37
dlPFC	L	–44	28	38	4.18
dmPFC	L	–4	54	42	3.97
Midcingulate gyrus	L	–2	–34	48	3.65
Postcentral gyrus	R	30	–46	66	7.2
Postcentral gyrus	L	–4	–50	70	3.46
**Coercive harming vs. Helping**					
Anterior insula	R	32	14	–20	5.59
Amygdala	L	–26	–8	–12	3.27
Hippocampus	L	–30	–14	–12	3.46
Fusiform	L	–26	–70	–10	4.1
Orbitofrontal cortex	L	–34	32	–8	4.01
Inferior temporal gyrus	L	–50	–66	–4	4.61
Anterior insula	L	–44	14	–2	4.15
dmPFC	R	6	64	28	4.99
Postcentral gyrus	L	–56	–24	28	5.58
Cuneus	L	–16	–86	36	4.25
dlPFC	L	–56	6	38	3.98
**Coercive helping vs. Harming**					
dlPFC	R	26	18	48	4.51
Middle frontal gyrus	R	36	54	2	3.77
Angular gyrus	R	42	–64	48	4.54
Posterior cingulate	R	8	–48	38	3.34
rTPJ	R	52	–50	20	3.26
**Placebo vs. Lorazepam**					
Hippocampus*	L	–28	–40	4	2.25*
dlPFC*	R	48	32	30	2.44*
**(Coercive Harming - Coercive Helping)| Placebo > (Coercive Harming – Coercive Helping)| Lorazepam**					
Hippocampus*	L	–32	–10	–18	2.18*
Amygdala*	R	30	0	–14	1.8*
**(Coercive Harming - Coercive Helping)| Lorazepam > (Coercive Harming - Coercive Helping) | Placebo**					
rTPJ*	R	56	–46	20	1.88*
dmPFC*	R	42	30	28	2.29*

Pooled group results for all participants (*N* = 77). All clusters are significant at voxel-wise FWE-corrected *P* < 0.05, except those marked with an asterisk, which are taken from *a priori* predefined ROIs and significant at uncorrected *P* < 0.05. R, Right; L, left; dlPFC, dorsolateral prefrontal cortex; dmPFC, dorsomedial prefrontal cortex; vmPFC, ventromedial prefrontal cortex; rTPJ, right temporoparietal junction.

*Brain area activation is statistically significant.

**FIGURE 2:**
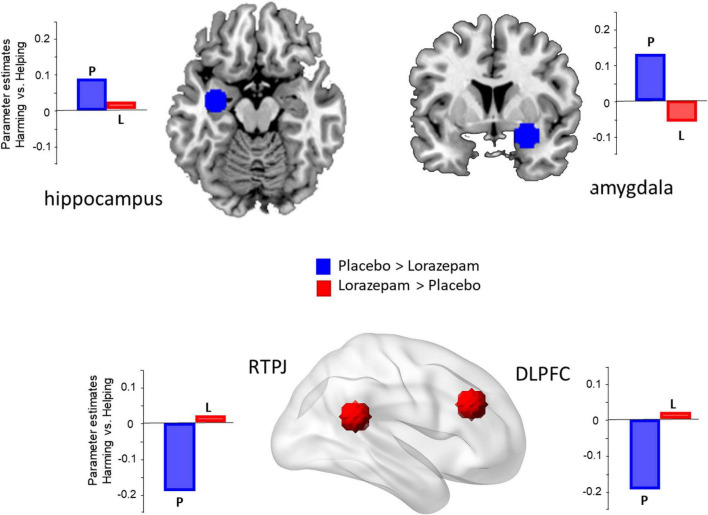
Lorazepam impact on the brain regions involved in coercive harming. The regions of interest (ROIs) included the amygdala (x 30, y 0, z –14), hippocampus (–32, –10, –18), rTPJ (56, –46, 20), and dlPFC (42, 30, 28). Acute lorazepam administration modulated the regions, depending on the factor of scenarios (harming vs. helping). The activity of harming vs. helping in the amygdala and hippocampus was reduced, whereas the activity in the dlPFC and rTPJ was increased.

Regarding the ROI results (Figure 2), significant interactions between administration (lorazepam vs. placebo) and scenario (harming vs. helping) were observed in the amygdala (*F*_1,76_ = 5.15, *P* = 0.026, η^2^ = 0.06), hippocampus (*F*_1,76_ = 4.89, *P* = 0.03, η^2^ = 0.06), and dlPFC (*F*_1,76_ = 5.87, *P* = 0.018, η^2^ = 0.07). The rTPJ had a marginal effect with a medium effect size (*F*_1,76_ = 3.70, *P* = 0.058, η^2^ = 0.05). The follow-up analyses indicated that the administration effect in the amygdala, hippocampus, dlPFC, and rTPJ had opposite directions depending on the factor of scenarios (coercive harming vs. helping). In the amygdala, harming induced a stronger neuro-hemodynamic responserelative to helping under the placebo condition (harming vs. helping: 0.179 ± 0.037 vs. 0.062 ± 0.034; *t*_76_ = 3.381, *Bonferroni-corrected P* < 0.05, Cohen’s *d* = 0.398), whereas no such difference was found in the lorazepam condition (harming vs. helping: 0.16 ± 0.046 vs. 0.164 ± 0.042; *t*_76_ = –0.109, *Bonferroni-corrected P* > 0.05). In the hippocampus, harming induced a stronger neuro-hemodynamic response relative to helping under the placebo condition (harming vs. helping: 0.135 ± 0.03 vs. 0.052 ± 0.023; *t*_76_ = 3.015, *Bonferroni-corrected P* < 0.05, Cohen’s *d* = 0.356), whereas no such difference was found in the lorazepam condition (harming vs. helping: 0.11 ± 0.028 vs. 0.107 ± 0.027; *t*_76_ = 0.115, *Bonferroni-corrected P* > 0.05). In the dlPFC, helping induced a stronger neuro-hemodynamic response relative to harming under the placebo condition (harming vs. helping: 0.112 ± 0.077 vs. 0.315 ± 0.085; *t*_76_ = –3.197, *Bonferroni-corrected P* < 0.05, Cohen’s *d* = 0.368), whereas no such difference was found in the lorazepam condition (harming vs. helping: 0.1 ± 0.091 vs. 0.093 ± 0.075; *t*_76_ = 0.124, *Bonferroni-corrected P* > 0.05). In the rTPJ, helping induced a stronger neuro-hemodynamic response relative to harming under the placebo condition (harming vs. helping: 0.02 ± 0.066 vs. 0.195 ± 0.075; *t*_76_ = –2.677, *Bonferroni-corrected P* < 0.05, Cohen’s *d* = 0.308), whereas no such difference was found in the lorazepam condition (harming vs. helping: 0.07 ± 0.076 vs. 0.061 ± 0.07; *t*_76_ = 0.129, *Bonferroni-corrected P* > 0.05).

**FIGURE 3:**
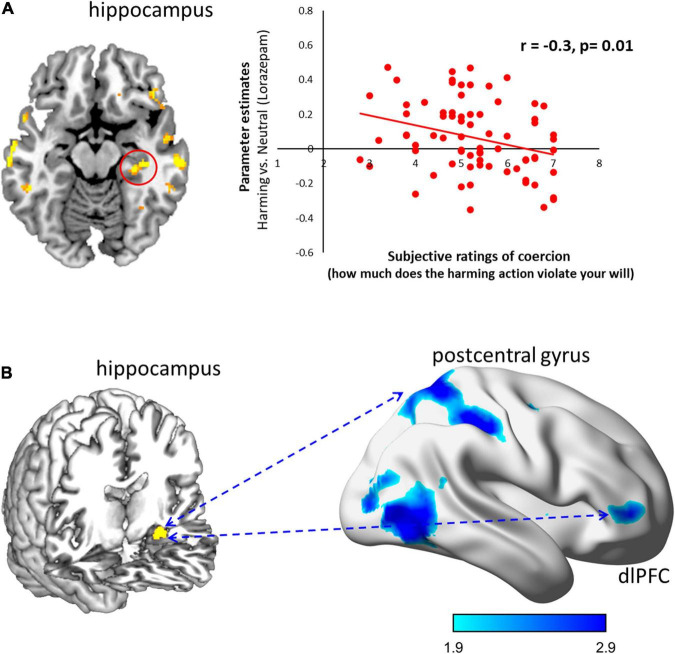
Lorazepam impact on the neural correlates and functional connectivity for subjective experience of coercive harming. **(A)** The subjective experience of coercion was assessed by the violation of free will. After lorazepam administration, less activity in the hippocampus (30, –28, –12) predicted higher subjective ratings of coercion (*r* = –0.3, *P* = 0.01). Clusters from the whole-brain contrast thresholded at *P* < 0.05 for viewing. **(B)** Lorazepam relative to placebo administration significantly reduced the coupling of the hippocampus (–30, –12, –18) with dlPFC (48, 48, 2), postcentral gyrus (14, –58, 64), and inferior temporal gyrus (54, –62, –10) in response to coercive harming. Results from the whole-brain contrast thresholded at *P* < 0.001 and cluster extent *k* > 10 for viewing.

To examine the association between the subjective experience of coercion and the observed neural responses, we did a whole-brain correlation analysis where subjective ratings were computed as a continuous variable with FWE rate at *P* < 0.05 (Figure 3A). After lorazepam administration, the activity in the hippocampus to coercive harming was significantly negatively correlated with subjective ratings of coercion (*r* = –0.3, *P* = 0.01).

### Functional connectivity

Lorazepam triggered distinct patterns in the functional couplings (Figure 3B). After lorazepam administration, the PPI analysis seeded in the hippocampus (–30, –12, –18) showed a significant negative coupling with the dlPFC (20, 22, 48; 40, 28, 52), orbitofrontal cortex (20, 36, –18), temporal pole (56, 8, –14), anterior cingulate cortex (4, 22, –6), postcentral gyrus (–50, –20, 58), superior temporal gyrus (–54, –26, 4), supplementary motor area (–10, –22, 50), medial frontal gyrus (20, 60, 0), and superior temporal gyrus (50, –22, 6). Whereas after placebo administration, the left hippocampus showed significantly positive connectivity with the dlPFC (52, 40, 2; –40, 44, 32), middle occipital gyrus (–44, –78, 4), middle frontal gyrus (–26, –2, 50), and supplementary motor area (–6, 4, 76). Importantly, lorazepam relative to placebo administration significantly decreased the couplings of the left hippocampus with the dlPFC (48, 48, 2), postcentral gyrus (14, –58, 64), and inferior temporal gyrus (54, –62, –10) during coercive harming.

## Discussion

With the present fMRI study, we aimed to elucidate the effects of the drug lorazepam on the sense of agency under coercion, as well as its neural underpinnings, by means of a virtual obedience paradigm. We found that RTs to initiate moral behaviors show an interaction effect with drug administration and scenario.

It is important to note that RTs may be considered an objective proxy measure for sedation, with the latter being a possible side effect of the GABA receptor agonist lorazepam (Vermeeren et al., 1995; Curran et al., 1998; Mintzer and Griffiths, 2003), and alongside psychomotor performance impairment (Smiley, 1987; Van Ruitenbeek et al., 2010). This makes it necessary to verify that any changes caused by lorazepam are not merely the results of such side effect. Our rationale for disentangling the effects of lorazepam due to sedation or due to its effect on emotional responses, was that if the RTs could be ascribed to the former, participants would exhibit increased RTs in the lorazepam administration condition independently of which moral valence they would be responding to. Here, the lorazepam effect was found to have opposite directions depending on the factor of moral valence.

When it comes to the sense of agency, previous literature uncovered explicit and implicit approaches to measure such a subjective phenomenon. The implicit index, as measured by the intentional binding effect, was based on the subjective perception of time compression between an action and its effects (Moore, 2016; Haggard, 2017); whereas for the explicit measures for the sense of agency, responsibility and guilt ratings were used (Caspar et al., 2018, 2020a). Nevertheless, the speed in which the decision of a moral action is made, as measured by RTs, could equally help illuminate an agent’s underlying moral character (Critcher et al., 2013). Agents who make an immoral decision quickly (versus slowly) are frequently evaluated more negatively by others. Conversely, agents who arrive at a moral decision quicker (versus slower) receive particularly positive moral character evaluations. Quicker decisions carry this signal value as they are assumed to indicate certainty of behavior, reflecting unambigious motives as backdrop drivers of such actions. On the contrary, the longer an agent takes to act during such task, the more mixed the agent’s motivations are assumed to be, hence the polarized moral character evaluations. Consequently, RTs may index the subjects’ implicit sense of agency or the willingness to conform to the instruction of an authority figure, whereas subjective ratings of coercion represent its explicit assessment. The results showed that, under the effects of lorazepam, RTs increased when harming others, but decreased when helping, despite of comparable self-reported ratings. It is reasonable to advance our hypothesis that the GABA agonist could help free participants from coercive control, enable them to recover their sense of agency, and follow their own will, thus slowing down their harming actions and accelerating those behaviors devoted for helping. Based on the interaction of administration and scenario observed through the RT data, where helping RTs were significantly shorter than both harming and neutral RTs after lorazepam administration, while harming RTs did not differ from the neutral RTs, we assume that increasing GABA activity via a low oral dose of a benzodiazepine (lorazepam, 0.5 mg) might exert a larger effect for helping actions, as compared to harming behaviors. As there were differing lorazepam dosages suggesting a modulation of neurobehavioral assessments among the literature, we decided to use a clinically non-effective dose of 0.5 mg lorazepam, for both safety reasons and to avoid any sedation effects (Arce et al., 2006; Diaper et al., 2012; Perkins et al., 2013; Gagnon et al., 2019). This might explain the significant interaction of lorazepam observed in the RTs without a main general slowing effect.

**FIGURE 4:**
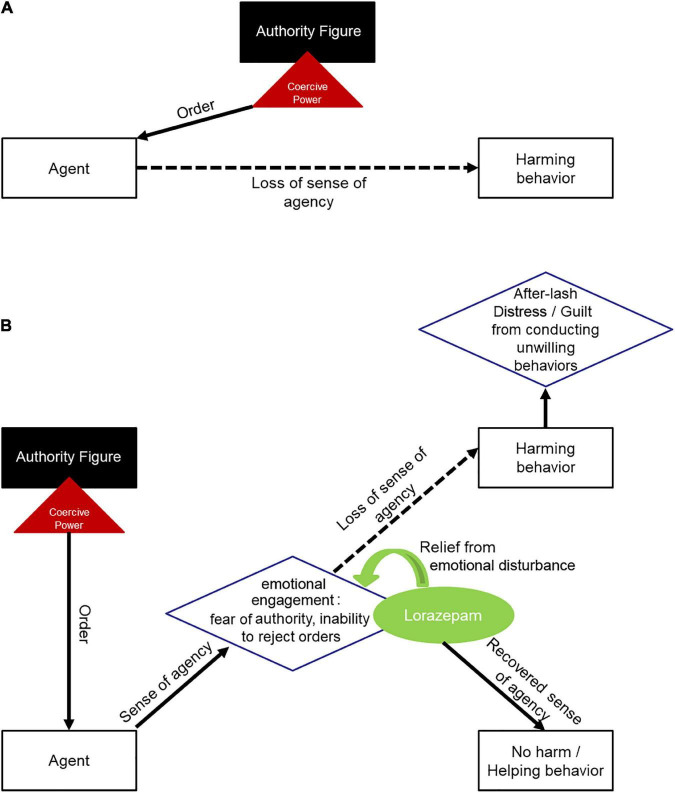
Framework models for obedience to authority under coercion. **(A)** Depicts previous model, where an agent is given a coercive order by an authoritative figure, which dilutes the agent’s sense of agency, leading the agent to perform a harmful act toward another person. **(B)** Depicts the new model, where an agent is given a coercive order by an authoritative figure, triggering emotional reactions in the agent and diminishing the agent’s sense of agency, thus, the agent proceeds to perform a harmful act toward another person. After lorazepam administration, the anxiolytic drug would reduce emotional engagement produced by coercion, letting the agent free cognitive power in order to reappraise its decisions and act with freedom from authority.

To explain the behavior of the agents under coercion, previous studies have set the players distributed in a rather simple model (Caspar et al., 2016, 2018): (a) an agent is given an order by an authority figure, (b) this order dilutes the agent’s sense of agency (as it is given by another person other than oneself), and (c) the agent performs a harmful behavior to another person (Figure 4A). We thus see necessary to put forward a new model (Figure 4B), where an authority figure will issue an order to an agent, which will trigger an emotional reaction (probably akin to anxiety) in the latter, as well as the corresponding loss of sense of agency, and making the agent execute a harmful act against another person. It is in this “emotional reaction” portion of the model where lorazepam would exert its action.

Alongside the above findings, we also observed that coercive harming versus helping recruited the activity in the amygdala and insula. This finding is in line with previous reports which posit that directly harming others generates an aversive emotional “gut” reaction (Greene et al., 2004). Conflicting with others is a common trigger for, or worsens anxiety-like negative emotional responses (Steimer, 2002; Grupe and Nitschke, 2013). Furthermore, and although Milgram’s accounts have been contested as of late, there were still participants that did believe they were delivering the shocks and that did not retaliate against the experimenter (Perry, 2013). In such cases, the experimenters’ authority during the experiments –based on French and Raven’s classic categories of the bases of social power (French and Raven, 1959)– was perceived not only as a form of expert and legitimate power, but also as coercive power (so much that there was no significant difference between the three) (Blass, 1999). As such, the experimenters were perceived as capable of administering punishment (threat) to the participants if they did not stick to their orders –constituting a source of negative emotionality.

Here, it is likely to make a case for lorazepam as a freer of cognitive power –letting participants to reason more clearly. Lorazepam has been suggested to increase ruthlessness, as it was observed that its administration drove participants to endorse harmful behaviors due to the drug’s ability to reduce threat intensity during moral dilemmas (Perkins et al., 2013). Furthermore, anxiolytic drugs are presumed to cause their effects by altering the emotional negativity accompanying anxiety and/or through cognitive modulation (Richter et al., 2010). Here, we did find that the lorazepam effect did not only decrease the activity in the amygdala and hippocampus, but also increased the activity in the dlPFC and rTPJ. The dlPFC has been consistently implicated in the cognitive control of motor behaviors, monitoring ongoing actions in alignment to internal goals (Miller, 2000; Morris et al., 2014). A number of meta-analyses have demonstrated rTPJ engagement in computational processes associated with sense of agency (Decety and Sommerville, 2003; Decety and Lamm, 2007). It is not surprising to see that lorazepam can increase the dlPFC and rTPJ activity, as a way to regain cognitive control and sense of agency, which, in turn, slows down coercive harming. Furthermore, the activation in the anterior insular cortex, midcingulate cortex, amygdala, and putamen during the unwilling harming condition, may suggest that the neural responses associated with empathy and guilt processing could be linked to the prolonged RTs or to less motivation for harming.

Notably, after lorazepam administration, the weaker activity in the hippocampus during coercive harming predicted higher subjective ratings of coercion (see Figure 3A). Based on a role-playing game, the two-dimensional geometric model of social relationships, a “social space” framed by power and affiliation, predicted the activity in the hippocampus, suggesting its critical involvement in a map for social navigation (Tavares et al., 2015). Moreover, we also found a negative coupling of the hippocampus with the dlPFC during coercive harming after lorazepam administration. Accordingly, the lorazepam effect could interfere with hippocampal activity in response to coercive harming. This would render participants to resist authority and fight against coercive control, in regards with more subjective feelings of coercion, and to express how much the action would violate their own will to a larger degree.

Some limitations of this study should be acknowledged. First, this study may have a confined ecological validity due to the use of a virtual obedience paradigm in its experimental design, as the paradigm could be affected by individual differences regarding the willingness to please the experimenter or to conduct voluntary harmful acts, and which may also affect moral motivation under coercion and the respective RTs. However, due to ethical concerns, as well as for neuroimaging purposes, this was the best course of action that could be thought of and consequently devised. While the successive image sequence for visual feedback was designed to help prime the participants as if they were being the agents of the simulated actions, it was inevitable to rule out the effects of the negative (or positive) affects that were caused by the aversive (or favorable) responses toward the perception of the following harming/helping pictures. However, these were similar to the affective responses incurred by coercion in a natural setting. The negative affect toward coercion was related to both the coercive power as well as the harming action *per se*. In real-life situations, individuals who obey coercive orders to harm exhibit differences in emotional responses to their immoral actions. Using the Nuremberg trials as a historical example, some war criminals being processed took their own lives out of guilt-like anxiety even before the trials began, whereas others attended the whole prosecution with seeming indifference (Fray et al., 1996), which suggests other factors at play (Cheng et al., 2021a). To dissociate the negative affect of coercion from the perception of aversive pictures (or harming actions *per se*), future studies adopting a better control condition are warranted, such as a control condition with instructions simply asking participants to press the button in order to view the following (harming) pictures, without specifically priming them to simulate the action. Second, post-session questionnaires to assess the perceived interpersonal behavior of the experimenter might be helpful to evaluate the effects of response expectancy to the intervention in drug-placebo studies (Gaab et al., 2019). Third, although RTs for moral actions (as mentioned above) may very well index an individual’s sense of agency, the relationship between these two elements warrants further investigation. In our case, the participants may or may not be aware of their delay on the unwilling harming actions or the lorazepam effect of RTs.

## Conclusion

In conclusion, the present findings –which incorporate multimodal indices, including functional neuroimaging, neuropharmacological intervention, and behavioral assessments– provide evidence to corroborate the notion that agents under coercive pressure may suffer from anxiety-like emotional negativity, and that the GABA receptor agonist lorazepam might help in unveiling the power of authority and assist in the emergence of prosocial behavior. Noteworthy, in real life scenarios, coercive orders to harm third parties could as well come with negative consequences for the self in case of disobedience. While we found the freeing effect of lorazepam on the sense of agency, lorazepam might also promote harming behaviors in moral-personal dilemmas out of selfish motivation (Perkins et al., 2013). Therefore, lorazepam might vary its effect as a function of the fear of negative consequences due to disobedience and the magnitude of selfish motivations in regards to it. This study sets the base for further research taking into consideration the victims living under serious and insidious coercive violence, e.g., the victims of occupational, domestic and intimate partner violence. By identifying key factors (which may very likely be remorse/anxious feelings resulting from the tug of war between the fear of authority and self-consciousness, as well as the fear of the negative consequences due to disobedience), such research could help turn over the toll of coercion.

## Materials and methods

### Participants

To estimate the sample size needed for this placebo-controlled, crossover design study, we implemented G*power 3.1 (Faul et al., 2009). Based on the effect size *f* (ranging from 0.2 to 0.22) for the primary fMRI outcomes found in previous fMRI literature using similar stimuli (Chen et al., 2016, 2020a), and in order to have 95% power, 70–84 participants would be required with a two-sided type I error of 0.05. Accordingly, 80 participants were enrolled, with one participant being excluded due to loss of follow-up, and other two participants being equally excluded due to excessive head motion (no. 62 and no. 37, please see Supplementary Figure 1 and Supplementary Table 1 for the results of sensitivity tests and the head motion descriptive statistics). The resulting 77 healthy volunteers (40 males), aged between 21 and 31 (23.5 ± 2.2) years, participated in the study after providing written informed consent. Participants were screened for major psychiatric illnesses (e.g., general anxiety disorder, major depressive disorder, etc.) by the Structured Clinical Interview for DSM-IV Axis I Disorders (SCID-I) (First and Gibbon, 2004), and excluded if there was evidence of any psychiatric disorder, or any comorbid neurological disorder (e.g., dementia, seizures, etc.), or any history of alcohol or substance abuse or dependence, including present or past episodes. Furthermore, participants who had any history of head injury or endocrinal disorder, including present or past episodes, were equally excluded from this study. All participants had normal or corrected-to-normal vision and were not taking any medication at the time of study. None of the female participants were taking oral contraceptives. This study was approved by the Ethics Committee (YM104041E) and conducted in accordance with the Declaration of Helsinki. Participants were recruited from local community colleges through printed advertisements. During the enrollment, the participants were told that this study was designed to determine whether the pill was effective in changing emotional rating (such as guilty ratings) after playing an interactive computerized animation program. However, in each experimental session (lorazepam vs. placebo), they were blinded for drug type.

### Procedures

In this double-blind, placebo-controlled, crossover design study, participants received a single 0.5-mg dose of lorazepam (ATIVAN) on one day, and a single dose of placebo (i.e., vitamin E) on another day. A crossover study is a longitudinal study in which subjects receive a sequence of different treatments. Both lorazepam and placebo were administered orally. The experimental sequence of lorazepam and placebo administration was counter-balanced between participants through a Latin square design, which randomizes through having equal number of AB (lorazepam-placebo) and BA (placebo-lorazepam) sequences. Thus, half of the participants went first through the lorazepam session, and half of them went first through the placebo session. To coincide with the pharmacokinetics of lorazepam (Kyriakopoulos et al., 1978), fMRI scanning and behavior assessment took place approximately 2-h after treatment administration.

In this study, we designed a virtual obedience paradigm inspired by prior studies on obedience to authority (Caspar et al., 2016, 2018, 2020a,2020b; Chen et al., 2020a), in which an experimenter ordered a subject to inflict harm to a third party (Figure 1A). During fMRI scanning, participants watched the first image of a morally laden mini clip, then they were ordered (ordered via textual instructions) to press a handheld button in order to initiate the successive image sequence which show the actions taken to full completion and that carry different moral consequences, including harming, neutral, or helping actions. More specifically, before each coerced action, participants were told to “press the button to harm others” for harming condition, “press the button to help others” for helping condition, and “press the button to start the action” for neutral action, respectively. In order to investigate whether the moral valence (harming vs. helping scenarios) interacted with placebo and lorazepam administrations on the feelings of coercion, after MRI scanning, participants were asked to undergo the same procedures that they did within the scanner and rate about how much the order to commit the action (coercion) would violate their own will. Five random trials from both harming and helping scenarios that had opposite moral valence were presented to participants again. Participants performed the task again and after each trail they rated their feelings of coercion. The ratings were on a 1–7 Likert scale, from “my will was not violated at all” to “my will was strongly violated.” Participants underwent the same experimental procedure in both placebo and lorazepam administrations with an at least 2-weeks washout. The order of placebo and lorazepam administrations was counterbalanced across participants. All participants confirmed that they were unaware regarding what treatment they had actually received during each task run. After completion of each task run (lorazepam vs. placebo), the participants were thoroughly debriefed, as to inform them the rationale behind the research and potential side effects of lorazepam.

### Visual stimuli

Forty-five validated animations from previous fMRI studies were presented (Chen et al., 2020a,b). Each animation was comprised of three images, with no duration limit set for the 1st image, but a 200 ms duration set for the 2nd image, and a 1,000 ms duration set for the 3rd image, and portraying the following scenarios: (1) a person who is alleviating physical pain from a suffering person (helping) (e.g., removing a rock on top of a crushed leg or hand), (2) a person who is taking an action to physically harm another person (harming), and (3) a baseline stimuli depicting a person carrying out an action that is irrelevant to another person (neutral). The faces of the protagonists were not visible to ensure that no emotional reactions could be seen by the participants. The order of scenarios was randomized across participants. Participants were explicitly primed to mentally simulate the agent, and forced to press the button to initiate the moral actions along with visual feedback of moral scenarios. More specifically, the participant would observe the first image of the animation, then would have to press the button to induce the remaining two images to play out.

### Functional magnetic resonance imaging acquisition, data processing and analysis

Participants underwent two sessions of fMRI scanning (placebo and lorazepam) in different days. Stimuli were presented with the E-prime software (Psychology Software Tools, Inc., Pittsburgh, PA, USA) and an MRI compatible goggle (VisualStim Controller, Resonance Technology Inc.) in a three-level within-subject design of moral scenarios (harming vs. neutral vs. helping).

The scanning followed a block design (22.9 ± 0.6 s ON/13.2 ± 4.4 s OFF) and had two runs. Each run consisted of six ON blocks (two harming, two helping, and two neutral scenarios) intermixed with six OFF blocks. Each ON block consisted of five trials, and five inter-stimulus intervals (duration 2,200-ms each) with a fixation cross presented against a gray background. While the ISI (interstimulus interval) was set as 2,200-ms, the duration of each fMRI regressor was modeled with each participant’s actual RTs. Because the RT varies across trials and participants, the modeled duration self-served as jittering in nature, leaving the average length of each ON block 2294 ± 598 ms (mean ± SD). The sequence of the scenarios (harming, helping, neutral) was pseudo-randomized within each run. The order of runs was counterbalanced across participants (Cheng et al., 2021b).

Scanning was performed on a 3T Siemens Magnetom Trio-Tim magnet. For functional changes, changes in blood oxygenation level-dependent (BOLD) T2* weighted MR signal were collected along the AC–PC plane using a gradient echo-planar imaging (EPI) sequence (TR = 2200 ms, TE = 30 ms, FOV = 220 mm, flip angle = 90°, matrix = 64 × 64, 36 transversal slices, voxel size = 3.4 mm × 3.4 mm × 3.0 mm, no gap). High-resolution structural T1-weighted images were acquired using a 3D magnetization-prepared rapid gradient echo sequence (TR = 2530 ms, TE = 3.5 ms, FOV = 256 mm, flip angle = 7°, slice thickness = 1 mm, matrix = 256 × 256, no gap) (Cheng et al., 2021b).

Functional images were processed with SPM12 (Wellcome Department of Imaging Neuroscience, London, UK) in MATLAB 9.0 (MathWorks Inc., Sherborn, MA, USA). Structural T1 images were coregistered to the mean functional images, and a skull-stripped image was created from the segmented gray matter, white matter, and Cerebrospinal Fluid (CSF) images. These segmented images were combined to create a subject-specific brain template. EPI images were realigned and filtered (128-s cutoff), then coregistered to these brain templates, normalized to Montreal Neurologic Institute (MNI) space, and smoothed (8 mm FWHM, full width at half maximum). The hemodynamic response function was time-locked to stimulus onset (Cheng et al., 2021b).

Data were input into a general linear model, with movement parameters as nuisance regressors. There was no significant difference in movement parameters between lorazepam and placebo. None of participants had movements greater than 2 mm of translation or 0.03 degrees of rotation (Supplementary Table 1). A two-stage general linear model was used to examine the effect size of each condition. At the first level analysis, three conditions (harming, helping, neutral) were modeled separately with a duration of the participant’s RT beginning at the onset of each ON block. The null event (fixation) was modeled with the duration 13.2 ± 4.4 s. Linear contrasts were applied to obtain parameter estimates. At the second-level analysis, images of parameter estimates from the first-level analysis (helping > neutral; harming > neutral) were collapsed into a repeated-measure factorial design with moral valence (helping vs. harming) and drug administration (lorazepam vs. placebo) as the within-subject variables. After the creation of an anatomically defined gray matter mask that was derived based on the MNI avg152T1 template, this gray matter mask was explicitly specified and applied to the whole brain analysis. Based on this gray matter mask and followed by the suggestions of using noise spatial auto-correlation function (Cox et al., 2017), family-wise error (FWE) rate at *P* < 0.05 was determined with a threshold at uncorrected *P* < 0.001, cut-off, *t* = 3.118, and cluster extent of at least 10 contiguous voxels, using a Monte Carlo simulation built in 3dClustSim: https://afni.nimh.nih.gov/pub/dist/doc/program_help/3dClustSim.html.

To elucidate the lorazepam effect, regions of interest (ROIs) analyses in limbic areas were conducted for bilateral amygdala and hippocampus (Schunck et al., 2010; Patin and Hurlemann, 2011). Beyond existing literature on emotional processing, there may be additional cortical regions, which are pivotal in moral reasoning, modulated by anxiolytics. The coordinates for the right temporoparietal junction (rTPJ, 56, –50, 18) and dlPFC (42, 30, 26) were determined on the basis of neuroanatomical atlases and meta-analyses (Lamm et al., 2011; Bzdok et al., 2012). ROI data are reported for significant contrast image peaks within 10 mm of these *a priori* coordinates. Data extraction for the ROI analyses was performed using the MarsBaR toolbox^1^ implemented in SPM12.

### Functional connectivity analysis

Based on our whole-brain results and prior studies (Arce et al., 2006; Schunck et al., 2010), the psychophysiological interaction (PPI) analysis was seeded in the left hippocampus (–30, –12, –18) to estimate how lorazepam administration altered the functional connectivity of the hippocampus during the unwilling coercive harming condition (harming vs. neutral). The time series of the first eigenvariates of the BOLD signal were temporally filtered, mean corrected, and deconvolved to generate the time series of the neuronal signal for the source region, i.e., the left hippocampus, as the physiological variable in the PPI. The PPI analysis assesses the hypothesis that the activity in one brain region can be explained by an interaction between cognitive processes and hemodynamic activity in another brain region. As the hippocampus was selected as the PPI source region, the physiological regressor was denoted by the activity in the left hippocampus. Coercive harming (harming vs. neutral) was the psychological regressor. The interaction between the first and second regressors represented the third regressor. The psychological variable was used as a vector coding for the specific task (1 for harming, –1 for neutral) convolved with the hemodynamic response function. The individual time series of the left hippocampus was obtained by extracting the first principle component from all raw voxel time series in a sphere (4 mm radius) centered on the coordinates of the subject-specific hippocampus activations. These time series were mean-corrected and high-pass filtered to remove low-frequency signal drifts. PPI analyses were then carried out for each subject by creating a design matrix with the interaction term, the psychological factor, and the physiological factor as regressors. PPI analyses were performed for each session separately (lorazepam and placebo) to identify brain regions showing significant changes in functional coupling with the hippocampus during coercive harming in relation to lorazepam administration. Subject-specific contrast images were then entered into random effects analyses at FWE of *P* < 0.05 (thresholded at *P* < 0.001, uncorrected, *k* = 10).

## Data availability statement

The raw data supporting the conclusions of this article will be made available by the authors, without undue reservation.

## Ethics statement

The studies involving human participants were reviewed and approved by the Ethics Committee of National Yang-Ming University (YM104041E). The patients/participants provided their written informed consent to participate in this study. Written informed consent was obtained from the individual(s) for the publication of any identifiable images or data included in this article.

## Author contributions

YC and CC conceived and conceptualized the study. Y-CC and CC collected and analyzed the data. YC, RM, and CC conducted the necessary literature reviews and drafted the first manuscript. Y-TF provided critical feedback and helped shape the manuscript. All authors contributed toward the revision and writing the final draft.

## Footnotes

1
http://marsbar.sourceforge.net/

## References

[B1] Arendt H. (1994). *Eichmann in Jerusalem: a report on the banality of evil* (Revised and enlarged edition). New York, NY: Penguin Books.

[B2] Milgram S. (1965). Some conditions of obedience and disobedience to authority. *Human Relations* 18 57–76.

[B3] Milgram S. (1963). Behavioral Study of Obedience. *J Abnorm Psychol* 67 371–378. 10.1037/h0040525 14049516

[B4] Perry G. (2013). *Behind the shock machine: The untold story of the notorious Milgram psychology experiments.* New York, NY: New Press.

[B5] Frank J. B. Rodowski M. F. (1999). Review of psychological issues in victims of domestic violence seen in emergency settings. *Emerg Med Clin North Am* 17 657–677, vii. 10.1016/s0733-8627(05)70089-4 10516845

[B6] Myhill A. Hohl K. (2019). The “Golden Thread”: Coercive Control and Risk Assessment for Domestic Violence. *J Interpers Violence* 34 4477–4497. 10.1177/0886260516675464 27807208

[B7] Zeigler-Hill V. Southard A. C. Archer L. M. Donohoe P. L. (2013). Neuroticism and negative affect influence the reluctance to engage in destructive obedience in the Milgram paradigm. *J Soc Psychol* 153 161–174. 10.1080/00224545.2012.713041 23484345

[B8] Maner J. K. Richey J. A. Cromer K. Mallott M. Lejuez C. W. Joiner T. E. (2007). Dispositional anxiety and risk-avoidant decision-making. *Personality and Individual Differences* 42 665–675.

[B9] Drake K. E. (2010). Interrogative suggestibility: Life adversity, neuroticism, and compliance. *Personality and Individual Differences* 48 493–498.

[B10] Allik J. McCrae R. R. (2004). Toward a geography of personality traits: Patterns of profiles across 36 cultures. *Journal of Cross-Cultural Psychology* 35 13–28.

[B11] Caspar E. A. Christensen J. F. Cleeremans A. Haggard P. (2016). Coercion Changes the Sense of Agency in the Human Brain. *Curr Biol* 26 585–592. 10.1016/j.cub.2015.12.067 26898470 PMC4791480

[B12] Caspar E. A. Cleeremans A. Haggard P. (2018). Only giving orders? An experimental study of the sense of agency when giving or receiving commands. *PLoS One* 13:e0204027. 10.1371/journal.pone.0204027 30256827 PMC6157880

[B13] Caspar E. A. Ioumpa K. Keysers C. Gazzola V. (2020a). Obeying orders reduces vicarious brain activation towards victims’ pain. *Neuroimage* 222 117251. 10.1016/j.neuroimage.2020.117251 32798682

[B14] Caspar E. A. Lo Bue S. Magalhaes De Saldanha da Gama P. A. Haggard P. Cleeremans A. (2020b). The effect of military training on the sense of agency and outcome processing. *Nat Commun* 11 4366. 10.1038/s41467-020-18152-x 32868764 PMC7459288

[B15] Caspar E. A. Beyer F. Cleeremans A. Haggard P. (2021). The obedient mind and the volitional brain: A neural basis for preserved sense of agency and sense of responsibility under coercion. *PLoS One* 16:e0258884. 10.1371/journal.pone.0258884 34710149 PMC8553174

[B16] Johnston G. A. (2013). Advantages of an antagonist: bicuculline and other GABA antagonists. *Br J Pharmacol* 169 328–336. 10.1111/bph.12127 23425285 PMC3651659

[B17] Jones M. S. Barth D. S. (2002). Effects of bicuculline methiodide on fast (>200 Hz) electrical oscillations in rat somatosensory cortex. *J Neurophysiol* 88 1016–1025. 10.1152/jn.2002.88.2.1016 12163550

[B18] Krishek B. J. Moss S. J. Smart T. G. (1996). A functional comparison of the antagonists bicuculline and picrotoxin at recombinant GABAA receptors. *Neuropharmacology* 35 1289–1298. 10.1016/s0028-3908(96)00089-5 9014144

[B19] Ueno S. Bracamontes J. Zorumski C. Weiss D. S. Steinbach J. H. (1997). Bicuculline and gabazine are allosteric inhibitors of channel opening of the GABAA receptor. *J Neurosci* 17 625–634. 8987785 10.1523/JNEUROSCI.17-02-00625.1997 PMC6573228

[B20] Bruyns-Haylett M. Luo J. Kennerley A. J. Harris S. Boorman L. Milne E. (2017). The neurogenesis of P1 and N1: A concurrent EEG/LFP study. *Neuroimage* 146 575–588. 10.1016/j.neuroimage.2016.09.034 27646129 PMC5312787

[B21] Gould R. A. Otto M. W. Pollack M. H. Yap L. (1997). Cognitive behavioral and pharmacological treatment of generalized anxiety disorder: A preliminary meta-analysis. *Behavior Therapy* 28 285–305. 10.1016/S0005-7894(97)80048-2

[B22] Perkins A. M. Leonard A. M. Weaver K. Dalton J. A. Mehta M. A. Kumari V. (2013). A dose of ruthlessness: interpersonal moral judgment is hardened by the anti-anxiety drug lorazepam. *J Exp Psychol Gen* 142 612–620. 10.1037/a0030256 23025561

[B23] Cheng Y. Chou J. Martinez R. M. Fan Y. T. Chen C. (2021a). Psychopathic traits mediate guilt-related anterior midcingulate activity under authority pressure. *Sci Rep* 11 14856. 10.1038/s41598-021-94372-5 34290344 PMC8295253

[B24] Davis M. Whalen P. J. (2001). The amygdala: vigilance and emotion. *Mol Psychiatry* 6 13–34. 10.1038/sj.mp.4000812 11244481

[B25] Etkin A. Klemenhagen K. C. Dudman J. T. Rogan M. T. Hen R. Kandel E. R. (2004). Individual differences in trait anxiety predict the response of the basolateral amygdala to unconsciously processed fearful faces. *Neuron* 44 1043–1055. 10.1016/j.neuron.2004.12.006 15603746

[B26] Chen Y. C. Chen C. Martinez R. M. Etnier J. L. Cheng Y. (2019). Habitual physical activity mediates the acute exercise-induced modulation of anxiety-related amygdala functional connectivity. *Sci Rep* 9 19787. 10.1038/s41598-019-56226-z 31875047 PMC6930267

[B27] Gagnon H. Simmonite M. Cassady K. Chamberlain J. Freiburger E. Lalwani P. (2019). Michigan Neural Distinctiveness (MiND) study protocol: investigating the scope, causes, and consequences of age-related neural dedifferentiation. *BMC Neurol.* 19:61. 10.1186/s12883-019-1294-6 30979359 PMC6460537

[B28] Arce E. Miller D. A. Feinstein J. S. Stein M. B. Paulus M. P. (2006). Lorazepam dose-dependently decreases risk-taking related activation in limbic areas. *Psychopharmacology* 189 105–116. 10.1007/s00213-006-0519-8 17016713 PMC2839080

[B29] Diaper A. Papadopoulos A. Rich A. S. Dawson G. R. Dourish C. T. Nutt D. J. (2012). The effect of a clinically effective and non-effective dose of lorazepam on 7.5% CO2-induced anxiety. *Human Psychopharmacology: Clinical and Experimental* 27 540–548. 10.1002/hup.2261 23027657

[B30] Curran H. V. Pooviboonsuk P. Dalton J. A. Lader M. H. (1998). Differentiating the effects of centrally acting drugs on arousal and memory: an event-related potential study of scopolamine, lorazepam and diphenhydramine. *Psychopharmacology (Berl)* 135 27–36. 10.1007/s002130050482 9489931

[B31] Mintzer M. Z. Griffiths R. R. (2003). Lorazepam and scopolamine: A single-dose comparison of effects on human memory and attentional processes. *Exp Clin Psychopharmacol* 11 56–72. 10.1037//1064-1297.11.1.56 12622344

[B32] Vermeeren A. Jackson J. L. Muntjewerff N. D. Quint P. J. Harrison E. M. O’Hanlon J. F. (1995). Comparison of acute alprazolam (0.25, 0.50 and 1.0 mg) effects versus those of lorazepam 2 mg and placebo on memory in healthy volunteers using laboratory and telephone tests. *Psychopharmacology (Berl)* 118 1–9. 10.1007/bf02245243 7597114

[B33] Smiley A. (1987). Effects of minor tranquilizers and antidepressants on psychomotor performance. *J Clin Psychiatry* 48 22–28. 2891686

[B34] Van Ruitenbeek P. Vermeeren A. Riedel W. J. (2010). Memory in humans is unaffected by central H1-antagonism, while objectively and subjectively measured sedation is increased. *Eur Neuropsychopharmacol* 20 226–235. 10.1016/j.euroneuro.2009.12.003 20083393

[B35] Haggard P. (2017). Sense of agency in the human brain. *Nat Rev Neurosci* 18 196–207. 10.1038/nrn.2017.14 28251993

[B36] Moore J. W. (2016). What Is the Sense of Agency and Why Does it Matter? *Front Psychol* 7:1272. 10.3389/fpsyg.2016.01272 27621713 PMC5002400

[B37] Critcher C. R. Inbar Y. Pizarro D. A. (2013). How quick decisions illuminate moral character. *Social Psychological & Personality Science* 4 308–315.

[B38] Greene J. D. Nystrom L. E. Engell A. D. Darley J. M. Cohen J. D. (2004). The neural bases of cognitive conflict and control in moral judgment. *Neuron* 44 389–400. 10.1016/j.neuron.2004.09.027 15473975

[B39] Grupe D. W. Nitschke J. B. (2013). Uncertainty and anticipation in anxiety: an integrated neurobiological and psychological perspective. *Nat Rev Neurosci* 14 488–501. 10.1038/nrn3524 23783199 PMC4276319

[B40] Steimer T. (2002). The biology of fear- and anxiety-related behaviors. *Dialogues Clin Neurosci* 4 231–249. 22033741 10.31887/DCNS.2002.4.3/tsteimer PMC3181681

[B41] French J. R. P. Jr. Raven B. (1959). “The bases of social power,” in *Studies in social power*, ed. Cartwright D. (Ann Arbor, MI: Institute for Social Research), 150–167.

[B42] Blass T. (1999). The Milgram Paradigm After 35 Years: Some Things We Now Know About Obedience to Authority. *Journal of Applied Social Psychology* 29 955–978. 10.1111/j.1559-1816.1999.tb00134.x

[B43] Richter A. Grimm S. Northoff G. (2010). Lorazepam modulates orbitofrontal signal changes during emotional processing in catatonia. *Human Psychopharmacology: Clinical and Experimental* 25 55–62. 10.1002/hup.1084 20041475

[B44] Morris R. W. Dezfouli A. Griffiths K. R. Balleine B. W. (2014). Action-value comparisons in the dorsolateral prefrontal cortex control choice between goal-directed actions. *Nat Commun* 5 4390. 10.1038/ncomms5390 25055179 PMC4124863

[B45] Miller E. K. (2000). The prefrontal cortex and cognitive control. *Nat Rev Neurosci* 1 59–65. 10.1038/35036228 11252769

[B46] Decety J. Sommerville J. A. (2003). Shared representations between self and other: a social cognitive neuroscience view. *Trends Cogn Sci* 7 527–533. 10.1016/j.tics.2003.10.004 14643368

[B47] Decety J. Lamm C. (2007). The role of the right temporoparietal junction in social interaction: how low-level computational processes contribute to meta-cognition. *Neuroscientist* 13 580–593. 10.1177/1073858407304654 17911216

[B48] Tavares R. M. Mendelsohn A. Grossman Y. Williams C. H. Shapiro M. Trope Y. (2015). A Map for Social Navigation in the Human Brain. *Neuron* 87 231–243. 10.1016/j.neuron.2015.06.011 26139376 PMC4662863

[B49] Fray W. C. Spar L. A. School Y. L. (1996). *The Avalon Project 1996*.*

[B50] Gaab J. Kossowsky J. Ehlert U. Locher C. (2019). Effects and Components of Placebos with a Psychological Treatment Rationale - Three Randomized-Controlled Studies. *Sci Rep* 9 1421. 10.1038/s41598-018-37945-1 30723231 PMC6363794

[B51] Faul F. Erdfelder E. Buchner A. Lang A. G. (2009). Statistical power analyses using G*Power 3.1: tests for correlation and regression analyses. *Behav Res Methods* 41 1149–1160. 10.3758/BRM.41.4.1149 19897823

[B52] Chen C. Decety J. Huang P. C. Chen C. Y. Cheng Y. (2016). Testosterone administration in females modulates moral judgment and patterns of brain activation and functional connectivity. *Human Brain Mapping* 37 3417–3430. 10.1002/hbm.23249 27145084 PMC6867570

[B53] Chen C. Martínez R. M. Cheng Y. (2020a). The key to group fitness: The presence of another synchronizes moral attitudes and neural responses during moral decision-making. *NeuroImage* 213 116732. 10.1016/j.neuroimage.2020.116732 32173411

[B54] First M. B. Gibbon M. (2004). “Personality assessment,” in *Comprehensive handbook of psychological assessment*, Vol. 2 eds Hilsenroth M. J. Segal D. L. (Hoboken, NJ: John Wiley & Sons Inc.), 134–143.

[B55] Kyriakopoulos A. A. Greenblatt D. J. Shader R. I. (1978). Clinical pharmacokinetics of lorazepam: a review. *J. Clin. Psychiatry* 39 16–23. 30762

[B56] Chen C. Martinez R. M. Chen Y. Cheng Y. (2020b). Pointing fingers at others: The neural correlates of actor-observer asymmetry in blame attribution. *Neuropsychologia* 136 107281. 10.1016/j.neuropsychologia.2019.107281 31770551

[B57] Cheng Y. Martínez R. Chen Y.-C. Fan Y.-T. Chen C. (2021b). GABA Boosts Relief from Coercive Power: an fMRI study. *Preprint* 10.21203/rs.3.rs-151432/v1

[B58] Cox R. W. Chen G. Glen D. R. Reynolds R. C. Taylor P. A. (2017). FMRI Clustering in AFNI: False-Positive Rates Redux. *Brain Connect* 7 152–171. 10.1089/brain.2016.0475 28398812 PMC5399747

[B59] Patin A. Hurlemann R. (2011). Modulating amygdala responses to emotion: evidence from pharmacological fMRI. *Neuropsychologia* 49 70 6–717. 10.1016/j.neuropsychologia.2010.10.004 20933529

[B60] Schunck T. Mathis A. Erb G. Namer I. J. Demazières A. Luthringer R. (2010). Effects of lorazepam on brain activity pattern during an anxiety symptom provocation challenge. *J Psychopharmacol* 24 701–708. 10.1177/0269881109104864 19460871

[B61] Bzdok D. Schilbach L. Vogeley K. Schneider K. Laird A. R. Langner R. (2012). Parsing the neural correlates of moral cognition: ALE meta-analysis on morality, theory of mind, and empathy. *Brain Struct Funct* 217 783–796. 10.1007/s00429-012-0380-y 22270812 PMC3445793

[B62] Lamm C. Decety J. Singer T. (2011). Meta-analytic evidence for common and distinct neural networks associated with directly experienced pain and empathy for pain. *Neuroimage* 54 2492–2502. 10.1016/j.neuroimage.2010.10.014 20946964

