# Supplementary material for: The anti-anxiety drug lorazepam changes implicit behaviors but not explicit evaluations of sense of agency under authoritative pressure: A functional magnetic resonance imaging study
Source: Front Psychol. 2022 Nov 21;13:991357. doi: 10.3389/fpsyg.2022.991357 (PMC9719977; doi:10.3389/fpsyg.2022.991357)
Supplement: Supplementary file 1 [file Data_Sheet_1.DOCX]

**SUPPLEMENTARY MATERIALS**

**Figure S1: Box and Whisker Plots indicating the outliers in head motion during lorazepam administration.**

**A**. Head displacement along the z axis.


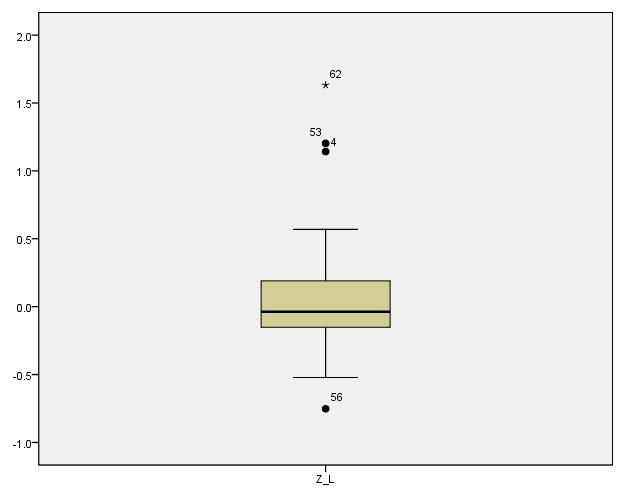


**B.** Roll movements of head rotation around the y axis.


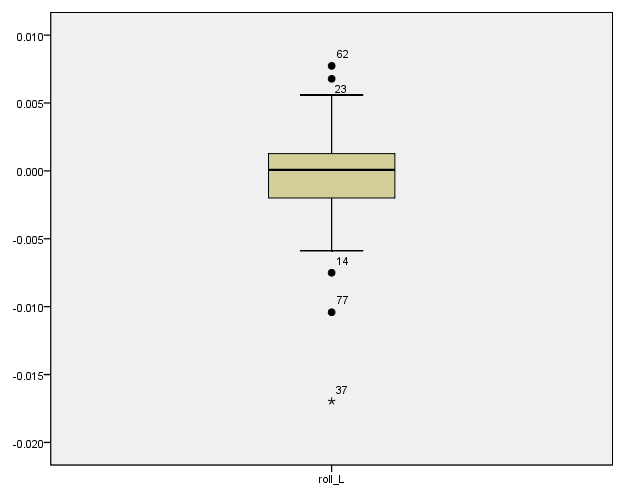


**Figure S2: Q-Q plots for reaction time (RT) data.**

**A.** Q-Q plots for harming RTs in the placebo condition.

**
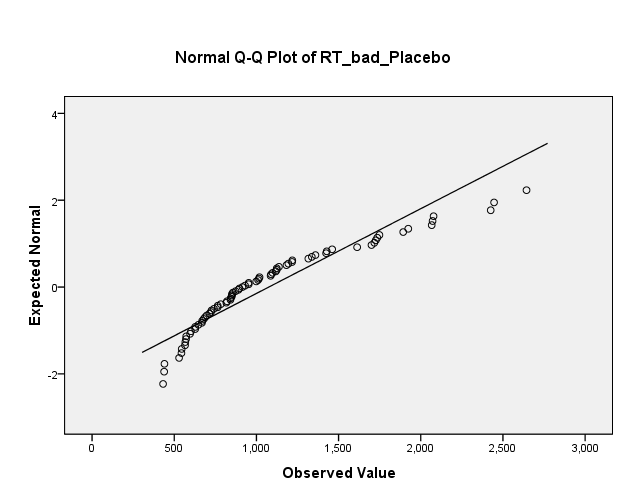

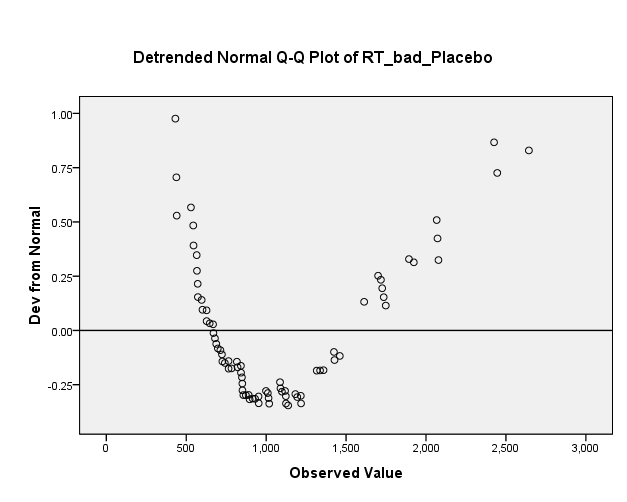
**

**B.** Q-Q plots for helping RTs in the placebo condition.

**
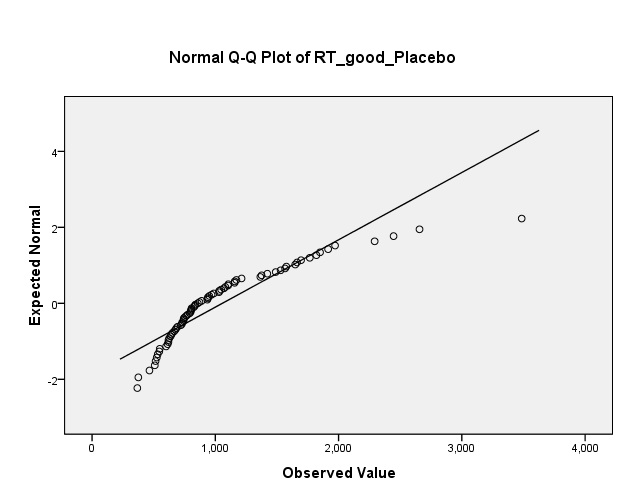

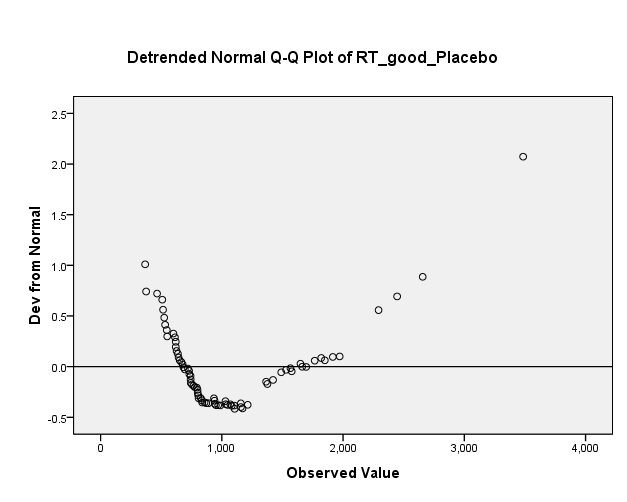
**

**C.** Q-Q plots for neutral RTs in the placebo condition.

**
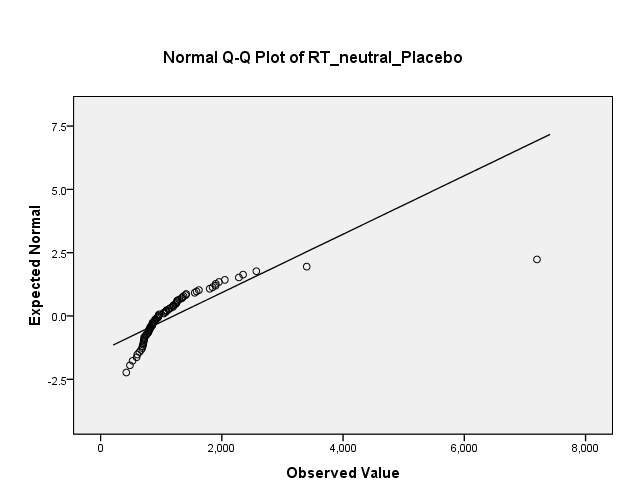

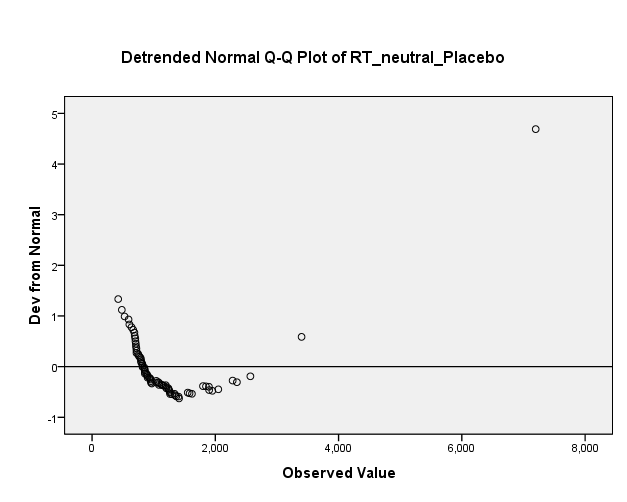
**

**D.** Q-Q plots for harming RTs in the lorazepam condition.

**
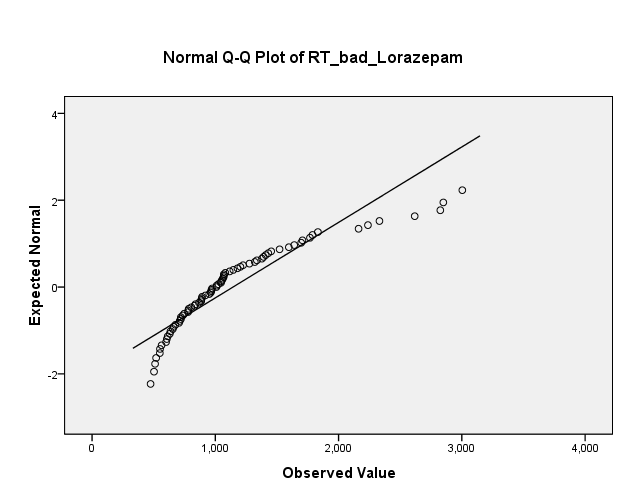

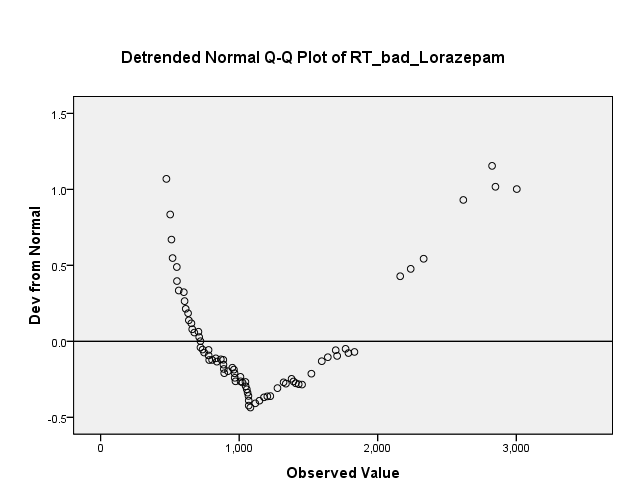
**

**E.** Q-Q plots for helping RTs in the lorazepam condition.

**
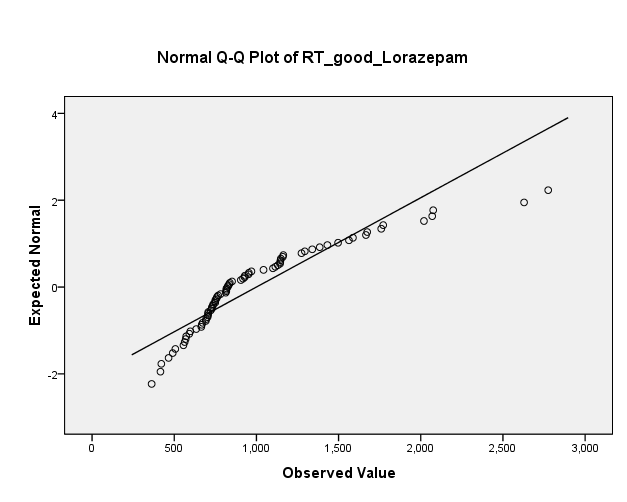

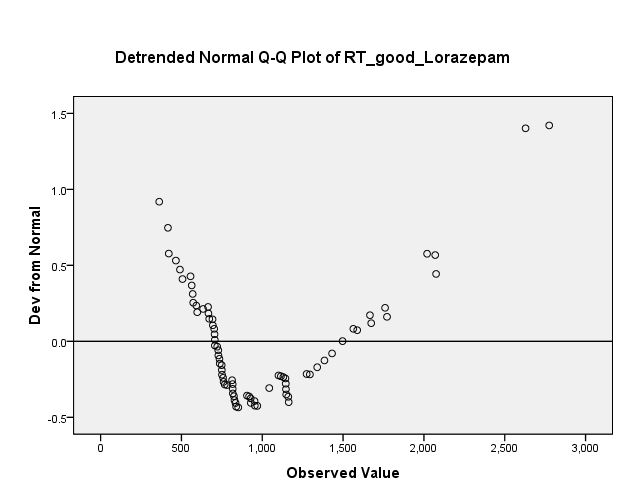
**

**F.** Q-Q plots for neutral RTs in the lorazepam condition.

**
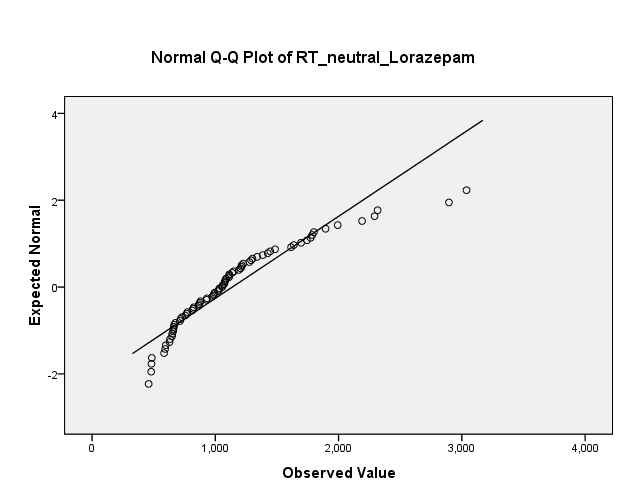

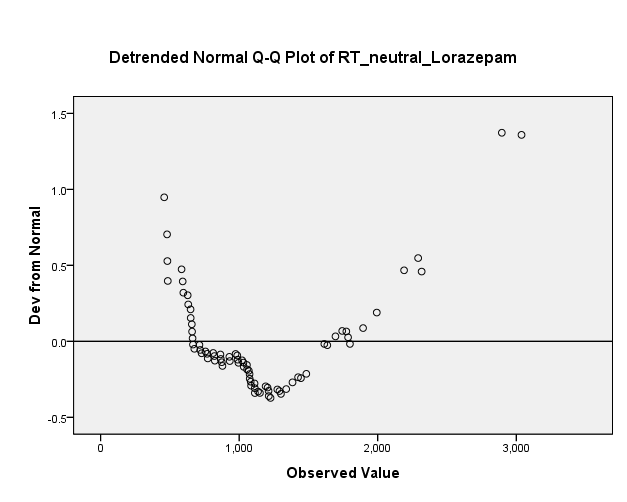
**

**Table S1:** **Participant head movements.** The six motion parameters (translation: x, y and z in mm and rotation: pitch, roll and yaw in degrees) were obtained from head movement correction for each participant during fMRI scanning.

| N=77 (after removing 2 outliers) | Lorazepam |  | Placebo |  |  |  |  |
| --- | --- | --- | --- | --- | --- | --- | --- |
| Motion  Parameters | Mean ± SE |  | Mean ± SE |  | T value | df | P value |
| x | 0.0399 ± 0.0134 |  | 0.036 ± 0.0133 |  | 0.22 | 76 | 0.827 |
| y | 0.1713 ± 0.0133 |  | 0.1495 ± 0.0126 |  | 1.378 | 76 | 0.172 |
| z | 0.0308 ± 0.0352 |  | -0.0285 ± 0.0337 |  | 1.525 | 76 | 0.131 |
| Pitch | -0.0009 ± 0.0009 |  | 0.0014 ± 0.0008 |  | -1.456 | 76 | 0.15 |
| Roll | -0.0004 ± 0.0003 |  | 0.0001 ± 0.0004 |  | -1.060 | 76 | 0.293 |
| Yaw | 0.0009 ± 0.0003 |  | 0.0013 ± 0.0004 |  | -0.924 | 76 | 0.358 |
| N=79 (without removing 2 outliers) |  |  |  |  |  |  |  |
| x | 0.0412 ± 0.0133 |  | 0.0354 ± 0.013 |  | 0.335 | 78 | 0.299 |
| y | 0.1703 ± 0.0133 |  | 0.1477 ± 0.0125 |  | 1.421 | 78 | 0.041 |
| z | 0.0474 ± 0.0401 |  | -0.0301 ± 0.0329 |  | 1.802 | 78 | 0.004 |
| Pitch | 0.0001 ± 0.0009 |  | 0.0014 ± 0.0008 |  | -1.275 | 78 | 0.044 |
| Roll | -0.0005 ± 0.0004 |  | 0.0001 ± 0.0004 |  | -1.149 | 78 | 0.062 |
| Yaw | 0.0009 ± 0.0003 |  | 0.0013 ± 0.0004 |  | -0.775 | 78 | 0.4 |

Notes: SE = standard error.

**Table S2:** **Normality tests for the raw reaction times (RTs) before log-transformation.**

|  | Kolmogorov-Smirnov^a^ | | |
| --- | --- | --- | --- |
|  | Statistic | df | Sig. |
| RT_harming_Placebo | .141 | 77 | .001 |
| RT_helping_Placebo | .167 | 77 | .000 |
| RT_neutral_Placebo | .223 | 77 | .000 |
| RT_harming_Lorazepam | .180 | 77 | .000 |
| RT_helping_Lorazepam | .179 | 77 | .000 |
| RT_neutral_Lorazepam | .148 | 77 | .000 |

**Table S3: Normality tests for reaction time (RT) differences before and after log-transformation.**

| **Before log** | Kolmogorov-Smirnov^a^ | | |
| --- | --- | --- | --- |
| **transformation** | Statistic | df | Sig. |
| RT_P_ harm vs neutral | .248 | 77 | .000 |
| RT_P_ help vs neutral | .230 | 77 | .000 |
| RT_L_ harm vs neutral | .173 | 77 | .000 |
| RT_L_ help vs neutral | .130 | 77 | .002 |
| **After log** | Kolmogorov-Smirnov^a^ | | |
| **transformation** | Statistic | df | Sig. |
| log_RT_P_ harm vs neutral | .076 | 77 | .200^*^ |
| log_RT_P_ help vs neutral | .047 | 77 | .200^*^ |
| log_RT_L_ harm vs neutral | .097 | 77 | .071 |
| log_RT_L_ help vs neutral | .066 | 77 | .200^*^ |

Abbreviations: P, placebo; L, lorazepam

**Complementary non-parametric analyses using raw RTs**

To examine whether there was a significant effect of scenario (harming vs. helping vs. neutral) after placebo and lorazepam administration, the raw RTs were subjected into non-parametric Friedman test for placebo and lorazepam administration, respectively. In the placebo condition, the main effect of scenario was significant (χ^2^ = 15.175, *P* = 0.001). Pairwise post hoc comparisons using Wilcoxon Signed Ranks Test showed that helping RTs were significantly shorter than neutral RTs (Z = -3.218, *Bonferroni-corrected P* < .05), while there were no significant differences found between helping and harming RTs (*Bonferroni-corrected P* > .05), and between harming and neutral RTs (*Bonferroni-corrected P* > .05). In the lorazepam condition, the main effect of scenario was significant (χ^2^ = 14.725, *P* = 0.001). Pairwise post hoc comparisons showed that helping RTs were significantly shorter than neutral RTs (Z = -2.978, *Bonferroni-corrected P* < .05) and harming RTs (Z = -3.381, *Bonferroni-corrected P* < .05), while there were no significant differences found between harming and neutral RTs (*Bonferroni-corrected P* > .05).
